# A patient with chronic sacroiliitis undiagnosed for three years after isotretinoin use

**DOI:** 10.1186/s12891-020-03290-6

**Published:** 2020-05-14

**Authors:** Cevriye Mülkoğlu, Barış Nacır

**Affiliations:** Department of Physical Medicine and Rehabilitation, Health Sciences University Ankara Training and Research Hospital, Ankara, Turkey

**Keywords:** Isotretinoin, Sacroiliitis, HLA-B27, Acne vulgaris

## Abstract

**Background:**

Isotretinoin (ISO) is a synthetic vitamin A derivative which has been used for treatment-resistant acne vulgaris. Although most musculoskeletal side effects of ISO are common, including myalgia, arthralgia, and back pain, sacroiliitis is one of the uncommon side effects. ISO-induced sacroiliitis usually completely resolves within a few months by the cessation of the drug.

**Case presentation:**

In this paper, we present a 26-year-old female patient with chronic sacroiliitis that was probably induced by ISO and not resolved by the discontinuation of the drug.

**Conclusion:**

In this patient, sacroiliitis was overlooked for three years. Therefore, ISO usage should be considered in the differential diagnosis of sacroiliitis and low back pain.

## Background

Acne vulgaris is a chronic inflammatory disease of the pilosebaceous unit in the skin. Increased sebum production, colonization of anaerobic *Propionibacterium acnes,* and impaired keratinization in follicles are some of the factors that play a role in the etiology of the disease [1]. Isotretinoin (ISO) is a synthetic vitamin A derivative that has been used for treatment-resistant acne vulgaris patients for about 40 years. The most common side effects associated with ISO are mucocutaneous and ocular reactions. Sacroiliitis is one of the uncommon adverse effects associated with ISO treatment. In a previous study, it was reported that the human leucocyte antigen B27 (HLA-B 27) positivity might predispose patients using ISO to the development of sacroiliitis [2]. Although the relationship between ISO and sacroiliitis has been demonstrated in the literature, the etiopathogenesis of the latter has not yet been completely elucidated. Our purpose was to assess the association between the two. In this paper, we present a case with chronic sacroiliitis which was triggered probably by ISO treatment and overlooked for 3 years.

## Case presentation

A 26-year-old woman was admitted to our outpatient clinic with the complaints of low back and right hip pain which had been present for 3 years. She reported to have early morning stiffness for about 40 min. The anamnesis revealed that she had taken a daily dose of 40 mg ISO for acne vulgaris for 8 months 3 years before, and then the drug was discontinued upon the recommendation of a dermatology doctor. When she presented to our outpatient clinic with the complaint of hip and back pain, she was no longer receiving ISO.

**Fig. 1:**
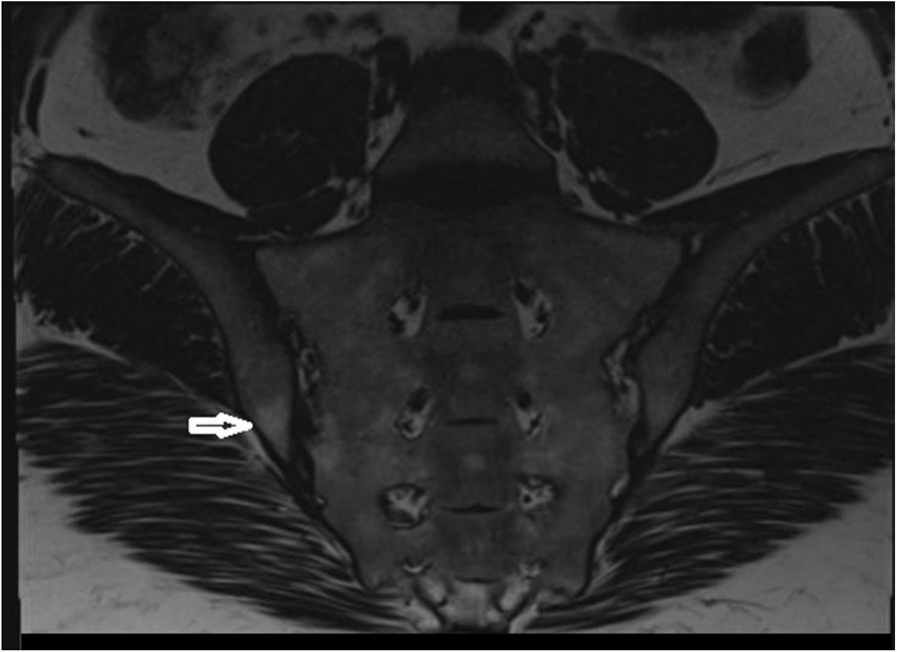
Coronal T1 image of sacroiliac joints. Arrow shows irregularity and subchondral sclerosis at the right side

**Fig. 2:**
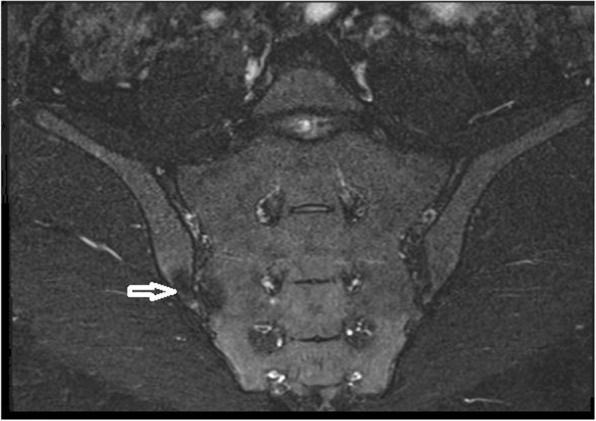
Coronal (fat suppressed) T2/STIR image of sacroiliac joints

The patient mentioned that she started to experience pain at the fourth months of ISO use, and she had no previous history of low back or buttock pain before this treatment. The pain in her back and right buttock sometimes also radiated to the thighs. Her back pain increased with rest and decreased with activity. She also had no history of infection that could cause reactive arthritis, psoriasis, uveitis, conjunctivitis or peripheral arthritis inconsistent with ankylosing spondylitis, enteropathic arthropathies and psoriatic arthropathy. She had no family history of axial spondyloarthropathy. On the physical examination, the range of lumbar flexion was limited and painful. It was found that the results of sacroiliac compression test and flexion-abduction-external rotation (FABER) test were positive for the right side. There was no peripheral arthritis or enthesopathy finding. The examination of other systems was unremarkable. In the blood test, HLA-B27 and anti-nuclear antibody were negative. C-reactive protein was 4.1 mg/L (normal range 0–5) and the erythrocyte sedimentation rate was 6 mm/hour. Other rheumatologic tests and cell blood count were unremarkable. Magnetic resonance imaging (MRI) revealed the presence of bilateral chronic sacroiliitis (Fig. 1-2). Cortical irregularity, erosions and subchondral changes were observed on the iliac surfaces adjacent to the joint bilaterally, especially the right side. There were oily changes in the opposite bone surfaces, more prominent at the right sacroiliac joint. No bone marrow edema was detected at the sacroiliac parts of the joints, consistent with chronic sacroiliitis. The patient was started on a daily dose of 120 mg acemetacin. At one-month follow-up, low back and hip pain was relieved and morning stiffness was decreased to 20 min. A home-based exercise program was added to the medical treatment. The patient was symptom-free after six months.

## Discussion and Conclusions

More severe forms of acne such as acne conglobata and acne fulminans are associated with musculoskeletal syndromes; however, acne vulgaris has no relationship with musculoskeletal symptoms [3]. Involvement of the sacroiliac joint has been reported in 21% of patients with acne fulminans accompanied by arthritis [4]. ISO is commonly used in the treatment of severe acne vulgaris. The musculoskeletal adverse effects of systemic ISO are common, and the most frequent rheumatic symptoms are musculoskeletal pain and arthralgia that occur in approximately 20% of the patients using ISO [5]. Sacroiliitis, enthesopathy, polyneuropathy, rhabdomyolysis, hyperostosis, and ligament calcification may also be seen, albeit rarely, due to ISO treatment. Sacroiliitis is a characteristic finding of ankylosing spondylitis and can be observed in other rheumatoid or non-rheumatoid diseases, such as psoriatic arthritis, familial Mediterranean fever, Behçet’s disease, and hyperparathyroidism. Our patient had no history of a rheumatologic or metabolic disease.

There are various studies on the prevalence of ISO-induced sacroiliitis. Selçuk et al. evaluated 73 patients with acne vulgaris who were receiving ISO to assess the prevalence of sacroiliitis in this patient group and found that the prevalence of ISO-associated inflammatory low back pain was 21.9% and that of sacroiliitis was 8.2% [1]. However, the authors did not evaluate HLA-B27. Another study included 42 patients using ISO and 32 patients using tetracycline for the treatment of acne vulgaris. There was unilateral sacroiliitis in only one patient in the ISO group (2.38%). No HLA-B27 positivity was observed in any patient in that study [6].

Sacroiliitis generally develops days or weeks after the beginning of ISO therapy. Sacroiliac pain may progress with mild or moderate acute phase elevation and findings of bone marrow edema in the sacroiliac joint on MRI. It is typically self-limited and resolves within months after the cessation of the drug. Glucocorticosteroids and non-steroidal anti-inflammatory drugs (NSAIDs) are effective in improving the symptoms. The mechanism involved in sacroiliitis induced by ISO has not yet been clearly explained. It is considered that the detergent-like characteristic of ISO alters the structure of the liposomal membrane and induces hypersensitivity reactions on the synovial cells which then become sensitive to degeneration with minor or mild trauma. This view is supported by cases of arthritis and sacroiliitis that develop during ISO therapy with an increase in exercise. Matrix metalloproteinases (MMP), activated by inflammatory cytokines, are known to be the reason for the destruction of the extracellular matrix in rheumatoid arthritis. Retinol and retinoic acid can stimulate MMP-2 activity. Since it is a derivative of retinoic acid, ISO activates MMP-2 activity and causes membrane damage in the joints [7, 8]. In previous studies on sacroiliitis conducted with patients receiving ISO for acne vulgaris, researchers were not able to conclusively relate the condition to either ISO or acne [2, 3, 7].

SAPHO (synovitis, acne, pustulosis, hyperostosis, osteitis) syndrome is a rare inflammatory condition with pustular skin disorders and osteoarticular inflammation. The axial skeleton (spine, sacroiliac joint) and peripheral bones may also be involved in SAPHO syndrome.

SAPHO syndrome and acne-related sacroiliitis usually do not respond to NSAIDs or simple analgesics. Therefore, systemic steroids or long-term combination therapy is required for a successful treatment [9]. Although SAPHO syndrome is classified among seronegative spondyloarthropathies, sacroiliitis is generally unilateral and accompanies hyperostosis, and its association with HLA-B27 is unknown [10]. It is possible that the use of synthetic retinoids may cause mesenchymal stem cell proliferation and differentiation to form osteoblasts in the entheses, leading to ossification, and prolonged retinoid therapy is associated with diffuse idiopathic skeletal hyperostosis [11]. Our patient had no hyperostosis according to the spine radiographs.

Koçak et al. reported 11 patients (three men and eight women) who had ISO-induced sacroiliitis. They evaluated these patients based on MRI findings. Sacroiliitis was found to have started within two months of treatment in six of these patients. MRI revealed mild sacroiliitis in five patients, moderate in three and severe in two. All 11 patients had bilateral sacroiliitis [12]. The complaints of our patient had started at the fourth month of ISO use. Our patient also had bilateral chronic sacroiliitis, which was more prominent at the right sacroiliac joint.

Sacroiliitis during ISO treatment typically improves once treatment is stopped, and it does not relapse [9]. However, in the current case, the cessation of ISO did not alleviate the symptoms.

The relationship between HLA-B27 positivity and ISO-induced sacroiliitis has not been fully understood. Ekşioğlu et al., who presented a case of isotretinoin-associated polyneuropathy and sacroiliitis, mentioned that HLA-B27-positive individuals might develop sacroiliitis [2]. However, in the literature, most cases of ISO-induced sacroiliitis were usually HLA-27 negative [12–15]. Similarly, in our patient, chronic sacroiliitis was accompanied by HLA-B27 negativity. Thus, it is possible to consider that there is no clear association between HLA-B27 positivity and sacroiliitis due to ISO.

Karadağ et al. evaluated four patients (all male) with ISO-induced sacroiliitis and detected bilateral sacroiliitis in three and left-sided sacroiliitis in one patient on MRI. HLA-B27 was negative for all patients. Once sacroiliitis was diagnosed, the authors stopped ISO treatment immediately and started all patients on sulfasalazine and indomethacin for treatment. The complaints of two patients were improved by this treatment within one month. However, in the remaining two cases, sacroiliitis did not resolve after six months of sulfasalazine treatment; thus, the medication was switched to adalimumab in one patient and methotrexate in the other. The sacroiliac MRI findings were normal for both patients at the ninth month of modified medical therapy. The authors stated that the severity of ISO-induced sacroiliitis varied from one patient to another [16]. Yilmaz Tasdelen et al. reported a 23 year-old male patient with bilateral arthritis of wrist and metacarpophalangeal joints when he was on isotretinoin treatment for cystic acne lesions. They administered indomethacine and the symptoms resolved completely. After 2 weeks stopping the indomethacine treatment, he presented with inflammatory back pain. Sacroiliac MRI showed an active inflammatory sacroiliitis on the left side. The patient was treated successfully with 10 mg prednisolone and 2 g/day sulfasalazine. At 6 months follow-up, control MRI revealed that no evidence of sacroiliitis [17]. We did not prescribe sulfasalazine to our patient because she had good response to acemetacin. We did not need to perform a follow-up MRI since the patient was symptom-free at the sixth-month of treatment and refused to undergo another MRI.

Coskun et al. presented two patients with ISO-induced bilateral active sacroiliitis and ISO-induced hidradenitis suppurativa. Despite the use of three different NSAIDs at maximum dose, the symptoms of the patients were not relieved. Therefore, biological treatment (infliximab, adalimumab) was started. In our patient, ISO treatment caused bilateral chronic sacroiliitis but not hidradenitis suppurativa. Our patient responded well to NSAID treatment (acemetacin 120 mg/day) within one month; therefore, we did not consider any biological drug for the continuation of treatment [18].

In patients with low back pain, ISO use should be questioned when considering a differential diagnosis of sacroiliitis; otherwise, sacroiliitis can be overlooked. Since the low back pain complaint of our patient had started four months after ISO use and had not resolved by the discontinuation of the drug, it is difficult to state that sacroiliitis definitely developed due to ISO treatment. However, we wanted to report this case due to a possible association between ISO and sacroiliitis.

In conclusion, although sacroiliitis is a rare adverse effect of ISO, patients with axial skeletal pain should be queried about the history of ISO use, and clinicians should consider the possibility of a relationship between ISO and sacroiliitis. Therefore, after the discontinuation of medication, patients suspected of sacroiliitis should be called for regular polyclinic visits and followed up with further imaging methods, such as MRI if necessary.

## Abbreviations

ISO: Isotretinoin
MRI: Magnetic resonance imaging
HLA-B27: Human leucocyte antigen B27
MMP: Matrix metalloproteinases
NSAIDs: Non-steroidal anti-inflammatory drugs
DISH: Diffuse idiopathic skeletal hyperostosis
SAPHO: Acne-related sacroiliitis and synovitis, acne, pustulosis, hyperostosis, osteitis

## References

[1] Baykal Selçuk L, Aksu Arıca D, Baykal Şahin H, Yaylı S, Bahadır S (2017). The prevalence of sacroiliitis in patients with acne vulgaris using isotretinoin. Cutan Ocul Toxicol.

[2] Eksioglu E, Oztekin F, Unlu E, Cakci A, Keyik B, Karadavut IK (2008). Sacroiliitis and polyneuropathy during isotretinoin treatment. Clin Exp Dermatol.

[3] Knitzer RH, Needleman BW (1991). Musculoskeletal syndromes associated with acne. Semin Arthritis Rheum.

[4] Geller AS, Alagia RF (2013). Sacroiliitis after use of oral isotretinoin-association with acne fulminans or adverse effect?. An Bras Dermatol.

[5] Kaplan G, Haettich B (1991). Rheumatological symptoms due to retinoids. Baillieres Clin Rheumatol.

[6] Alkan S, Kayiran N, Zengin O (2015). Isotretinoin-induced Spondyloarthropathy-related symptoms: a prospective study. J Rheumatol.

[7] Dincer U, Cakar E, Kiralp MZ (2008). Can isotretinoin induce sacroiliitis: three cases. Turk J Rheumatol.

[8] Levinson M, Gibson A, Stephenson G (2012). Sacroiliitis secondary to isotretinoin. Australas J Dermatol.

[9] Rozin A (2009). SAPHO syndrome: is a range of pathogen-associated rheumatic diseases extended?. Arthritis Res Ther.

[10] Zimmermann P, Curtis N (2016). Synovitis, acne, pustulosis, hyperostosis, and osteitis (SAPHO) syndrome - a challenging diagnosis not to be missed. J Inf Secur.

[11] Zhao S, Goodson NJ. Diffuse idiopathic skeletal hyperostosis and isotretinoin in cystic acne. BMJ Case Rep 2015;2015. pii: bcr2015209775. doi: 10.1136/bcr-2015-209775. 10.1136/bcr-2015-209775 PMC4480141 26106176

[12] Kocak O, Kocak AY, Sanal B, Kulan G (2017). Bilateral Sacroiliitis confirmed with magnetic resonance imaging during IsotretinoinTreatment: assessment of 11 patients and a review of the literature. Acta Dermatovenerol Croat.

[13] Dawoud NM, Elnady BM, Elkhouly T, Yosef A (2018). Adalimumab as a successful treatment for acne fulminans and bilateral acute sacroiliitis with hip synovitis complicating isotretinoin therapy. Indian J Venerol Leprol.

[14] Bachmeyer C, Charoud A, Turc Y, Callot V, Blum L, Aractingi S (2003). Isotretinoin-induced bilateral sacroiliitis. Dermatology.

[15] Aydog E, Ozturk G, Comert A, Tasdelen N, Akin Ö, Geler KD (2019). Sacroiliitis during isotretinoin treatment: causal association or coincidence?. North Clin Istanb.

[16] Karadağ ŞG, Sönmez HE, Tanatar A, Çakan M, Aktay AN (2020). Isotretinoin-induced sacroiliitis: case series of four patients and a systematic review of the literature. Pediatr Dermatol.

[17] Yilmaz Tasdelen O, Yurdakul FG, Duran S, Bodur H (2015). Isotretinoin-induced arthritis mimicking both rheumatoid arthritis and axial spondyloarthritis. Int J Rheum Dis.

[18] Coskun BN, Yagiz B, Pehlivan Y, Dalkilic E (2019). Isotretinoin-induced sacroiliitis in patients with hidradenitis suppurativa: a case-based review. Rheumatol Int.

